# Synthesis and Characterization of Antibiotic–Loaded Biodegradable Citrate Functionalized Mesoporous Hydroxyapatite Nanocarriers as an Alternative Treatment for Bone Infections

**DOI:** 10.3390/pharmaceutics14050975

**Published:** 2022-04-30

**Authors:** Nasser H. Alotaibi, Muhammad Usman Munir, Nabil K. Alruwaili, Khalid Saad Alharbi, Ayesha Ihsan, Alanood S. Almurshedi, Ikram Ullah Khan, Syed Nasir Abbas Bukhari, Mubashar Rehman, Naveed Ahmad

**Affiliations:** 1 Department of Clinical Pharmacy, College of Pharmacy, Jouf University, Sakaka 72388, Saudi Arabia; nhalotaibi@ju.edu.sa; 2 Department of Pharmaceutical Chemistry, College of Pharmacy, Jouf University, Sakaka 72388, Saudi Arabia; sbukhari@ju.edu.sa; 3 Department of Pharmaceutics, College of Pharmacy, Jouf University, Sakaka 72388, Saudi Arabia; nkalruwaili@ju.edu.sa; 4 Department of Pharmacology, College of Pharmacy, Jouf University, Sakaka 72388, Saudi Arabia; kssalharbi@ju.edu.sa; 5 National Institute for Biotechnology and Genetic Engineering College, Pakistan Institute of Engineering and Applied Sciences (NIBGE-C, PIEAS), Faisalabad 38000, Pakistan; aishaehsan@gmail.com; 6 Department of Pharmaceutics, College of Pharmacy, King Saud University, Riyadh 11451, Saudi Arabia; marshady@ksu.edu.sa; 7 Department of Pharmaceutics, Faculty of Pharmaceutical Sciences, Government College University Faisalabad, Faisalabad 38000, Pakistan; ikramglt@gmail.com; 8 Department of Pharmacy, Quaid-i-Azam University, Islamabad 45320, Pakistan; mrehman@qau.edu.pk

**Keywords:** hydroxyapatite, nanocarrier, antibiotic, multidrug resistance, bone infection, bacterial resistance, calcium phosphate

## Abstract

The continuing growth of bacterial resistance makes the top challenge for the healthcare system especially in bone-infections treatment. Current estimates reveal that in 2050 the death ratio caused by bacterial infections can be higher than cancer. The aim of this study is to provide an alternative to currently available bone-infection treatments. Here we designed mesoporous hydroxyapatite nanocarriers functionalized with citrate (Ctr–mpHANCs). Amoxicillin (AMX) is used as a model drug to load in Ctr–mpHANCs, and the drug loading was more than 90% due to the porous nature of nanocarriers. Scanning electron microscopy shows the roughly spherical morphology of nanocarriers, and the DLS study showed the approximate size of 92 nm. The Brunauer–Emmett–Teller (BET) specific surface area and pore diameter was found to be about 182.35 m^2^/g and 4.2 nm, respectively. We noticed that almost 100% of the drug is released from the AMX loaded Ctr–mpHANCs (AMX@Ctr–mpHANCs) in a pH-dependent manner within 3 d and 5 d at pH 2.0 and 4.5, respectively. The sustained drug release behaviour was observed for 15 d at pH 7.4 and no RBCs hemolysis by AMX@Ctr–mpHANCs. The broth dilution and colony forming unit (CFU) assays were used to determine the antimicrobial potential of AMX@Ctr–mpHANCs. It was observed in both studies that AMX@Ctr–mpHANCs showed a significant reduction in the bacterial growth of *S. aureus*, *E. coli*, and *P. aeruginosa* as compared to Ctr–mpHANCs with no bacteria-killing. Thus, we proposed that Ctr–mpHANCs can be used as a drug carrier and a treatment option for bone infections caused by bacteria.

## 1. Introduction

The treatment of bone infections (BIs) is a dynamic task to treat bone and joint ailments because their treatment costs billions of dollars annually [1]. BIs are mainly affected by bacterial attacks like *Staphylococcus aureus* (*S. aureus*), *Escherichia coli* (*E. coli*) with positive *β lactamase*, and *Pseudomonas aeruginosa* (*P. aeruginosa*) as in the case of osteomyelitis, bone injury, and osteoarthritis [2]. The antibiotics are used irrationally, and it directs the bacteria to mature and progress the resistance against available market antibiotics. One of the significant drastic and emerging issues globally is bacterial multidrug resistance (MDR) that is predominant due to biofilm formation, progressive changes in the normal genes to resistant ones, drug target site modification, antiobiotic modification, swarming, and elimination of the drug by efflux pump [3].

Many antibiotics got resistance due to biofilm formation property of bacteria and it is very challenging to treat biofilm related infections. These biofilms protect the bacteria, reduces the antibiotic action and causes the chronic infection to patients. According to reports, the biofilm formation makes the bacteria 1000 times more resistant against antibiotics in comparison to bacteria with no biofilm formation [4,5]. Furthermore, the antibiotics face difficulty to invade bacteria due to extracellular polymeric substances (EPS). This is the reason the bacteria are sheltered from the body’s immune system and cause resistance to the drug. This phenomenon has a significant impact on the action of antibiotics against microbes because these drugs cannot capture the bacteria due to biofilm, formation. These factors collectively provide the hurdles for the treatment of bone infections [6].

Due to MDR in bacteria, a patient has to take many combinations of medicines to get rid of the infection, causing the treatment to be toxic and more expensive. In some cases, even these regimens did not cure bacterial diseases due to advanced MDR [7]. Currently, we have fewer possibilities to treat such infections; hence, new active agents should be discovered to abolish the pathetic concern of MDR. However, it costs too much to develop a single new drug moiety in this era of economic complications, and scientists face funding issues.

Nanotechnology has proved to be a promising area to handle the challenge by developing nanosized materials that could address these threats to public health [3,8]. To treat such bone ailments, we should focus on a sustained drug delivery system that helps deliver the drugs to the target site of infection. Nanocarriers (NCs) with good biocompatible and decent biodegradable attitudes are preferred to use for that purpose [9]. Recently, scientists have developed augmented research taste in inorganic nanocarriers (IoNCs) as drug delivery carriers. These nanocarriers can be adjusted to fight against an anti-biofilm activity with extended delivery of the drugs and macromolecules [10]. IoNCs have numerous benefits compared to the NC system of polymers and organic nanoparticles due to minimal toxic problems, multi-functionality, and lesser immune response [11]. Contrasting the liposomal carrier system, IoNCs have shown resistance and stability to lipase and bile salts, respectively [12].

Hydroxyapatite materials (HAMs) have a resemblance with bone composition and are an excellent candidate to treat BIs [13]. These materials are a critical inorganic constituent of the teeth and bone in the vertebrate species. Additionally, they are valuable biomaterials with superb biocompatibility, nontoxic behaviour, good absorbability, high surface area, enhanced stability, and bone conductibility. The drugs can be carried out by these nanomaterials either by adsorbing on their surface or loading [14,15]. Furthermore, the porous nature of HAMs supports bone renovation with the sustained release of drug molecules [16]. These nanomaterials are widely used in many areas of biomedical research, for instance, protein adsorption, drug delivery, tooth and bone healing, water treatment, and antimicrobial application [17]. This is why HAMs have received much consideration from various scientists globally and have been examined broadly for applications in numerous research fields.

Amoxicillin (AMX), a beta-lactam antibiotic, is a broad-spectrum antibiotic that destroys gram-positive bacteria with narrow-spectrum against gram-negative bacteria. Its pharmacological activity is not enough to eradicate the beta-lactamase bacteria because it is unable to resist beta-lactamases. Because of this, AMX is given in combination with clavulanic acid to boost the susceptibility to microbes [18]. AMX is frequently used in bone infections, sinusitis and bronchitis, infections of the urinary tract, and typhoid. It has a short half-life of 1 h approximately. The different species of bacteria like *S. aureus*, *Klebsiella pneumonia*, *Enterobacter* species, *P. aeruginosa*, and *E. coli* have developed resistance to AMX [19].

This study is designed to synthesize the citrate-based functionalized mesoporous hydroxyapatite nanocarriers (Ctr–mpHANCs) and explore their future use to deliver AMX against MDR bacteria. Citric acid is used to functionalize the surface of mpHANCs as it provides numerous chemisorption sites for AMX to make complexes [20]. We optimize the pore size of mpHANCs, AMX loading and pH-sensitive drug release. In addition, we noted the pharmacological activity of the formulations against MDR strains of *P. aeruginosa*, *MRSA*, and *E. coli.*

## 2. Materials and Methods

Calcium chloride, sodium carbonate, citric acid, and phosphoric acid were purchased from Acros Organics (Geel, Belgium). Ammonium solution and Muller Hinton media were procured from Merck (Darmstadt, Germany), while diammonium hydrogen phosphate was purchased from Sigma-Aldrich (St. Louis, MO, USA). Sodium bicarbonate was purchased from Fisher Scientific Company (Waltham, MA, USA). All the materials were of the analytical grade. The local source provided amoxicillin as a gift sample. In addition, we obtained clinical strains of *MRSA*, *P. aeruginosa*, and *E. coli* from a research-based institute, the National institute for biotechnology and genetic engineering (NIBGE) Faisalabad, Pakistan.

### 2.1. Synthesis of Citrate Functionalized Mesoporous Hydroxyapatite Nanocarriers (Ctr–mpHANCs)

The synthesis of Ctr–mpHANC was carried out by co-precipitation method. Calcium chloride (CaCl_2_), sodium carbonate (Na_2_CO_3_), and phosphoric acid (H_3_PO_4_) were used as a calcium, carbonate, and phosphate source. Citric acid was used as a citrate source to disperse the particles in the formulation. An equal volume of the precursors CaCl_2_ and Na_2_CO_3_ were mixed on a magnetic stirrer for 1 h at 90 °C until the formation of a turbid solution indicating CaCO_3_ nanoparticles. Then citric acid was added to the above mixture to prepare the citrate stabilized CaCO_3_ nanoparticles during continuous stirring at a rate of 2 mL/min. The pH of the solution was maintained to 10 with an ammonia solution (25%). This magnetic stirring was continued, and H_3_PO_4_ was added to Ctr–CaCO_3_ nanocarriers dropwise to make citrate functionalized hydroxyapatite nanocarriers. Acetic acid was used as a pore-forming agent and to expel out residual carbonate from the formulation to produce Ctr–mpHANCs. Hong et al. reported that acetic acid acts as a pore-forming agent and the mesoporous structure is formed [21]. The mesoporosity is confirmed by the BET analysis. The prepared formulation was washed with ethanol three times to remove the acetic acid residues and then washed with deionized water five times. Finally, the prepared Ctr–mpHANCs were dried in a vacuum oven at 60 °C. Sole or non-functionalized hydroxyapatite nanocarriers were also prepared without adding the citrate ions for comparison purposes.

### 2.2. Preparation of AMX Loaded Ctr–mpHANCs

We used different concentrations of AMX (20, 30, and 50 mM) to load in mpHANCs to check the encapsulation efficiency. To prepare these, mpHANCs of 20 mg was added to each beaker, and 20 mM, 30 mM, and 50 mM AMX were placed in the respective beaker with formulation codes AMX1@Ctr–mpHANCs, AMX2@Ctr–mpHANCs, and AMX3@Ctr–mpHANCs, respectively. These mixtures were stirred for 2 h so that AMX could be loaded or adsorbed uniformly on mpHANCs. Then, the pellet was collected by centrifugation of all formulations at 13,000 rpm for 15 min. The residues were washed with deionized water to remove any physical adsorption of AMX on nanocarriers because it may give false encapsulation efficiency, and, finally, drying was done in a vacuum oven at 70 °C.

### 2.3. Estimation of Microbial Culture Resistance

We evaluated different antibiotic discs for their activity against specific microbial cultures by the disk diffusion method. This step was performed to choose the antibiotic which is resistant to all bacterial strains. The antibiotics we used include cefotaxime (CTX), Ceftriaxone (CRO), Aztreonam (ATM), Amoxicillin (AMX), Augmentin (AMC), Imipenem (IPM), Ceftazidime (CAZ), and Vancomycin (VA).

### 2.4. Characterization

The morphology of mpHANCs was observed by FE-SEM (JSM-7500 F, Jeol Ltd., Akishima, Tokyo, Japan), and the sample was prepared by casting a drop of mpHANCs on the silicon wafer. We checked the porosity and specific surface area with Micromeritics N_2_ adsorption/desorption (ASAP 2020, Micromeritics, Norcross, GA, USA). Subsequently, degassing measurement was achieved at 77 K. At the same time, Brunauer–Emmett–Teller (BET) was used to estimate the specific surface area of mpHANCs by using the values of adsorption at a relative pressure (p/p°) range from 0–0.95. In contrast, the distribution of pore volume and pore size were determined from the desorption curve by applying the model of Barrett Joyner–Halenda (BJH). Malvern Nano ZS was used to determine the particle size and zeta potential. The interaction of drug-nanocarrier was evaluated by ATR-FTIR (Bruker-Alpha, Karlsruhe, Germany). The scans of functionalized and non-functionalized mpHANCs were taken from 4000 cm^−1^ to 500 cm^−1^. This study was performed to check the compatibility of our formulations. The crystalline phase of our formulation was assessed by the X-ray diffraction (XRD) technique. The peaks were noted with scanning interval (2θ) ranging from 20° to 60° (D8 advances diffractometer by Bruker, Germany, coupled with Cu Kα radiation and λ = 0.1540 nm). XRD coupled with Cu Kα is used because wavelength of copper K-alpha radiation is intense, monochromatic and is of the order of the lattice spacing found in crystalline solids to produce efficient diffraction pattern.

#### 2.4.1. Physical State and Thermal Behaviour of Amoxicillin Inside Ctr–mpHANCs

The physical state of AMX in our formulation, either amorphous or crystalline, is determined by differential scanning calorimetry (DSC) within a temperature range of 25–500 °C at a heating rate of 20 °C/min under a nitrogen purge of 30 mL/min. In addition, thermogravimetric analysis (TGA, SDT Q600) was performed to determine the thermal stability of the sole and drug-loaded Ctr–mpHANCs. We used the temperature specification from 25 °C to 600 °C with a 10 °C/min ramp.

#### 2.4.2. Encapsulation Efficiency Determination

An indirect method was used in determining the encapsulation efficiency (EE) of all AMX@Ctr–mpHANCs. First, the three different concentrations of AMX@Ctr–mpHANCs were centrifuged separately at 13,000 rpm for 12 min, and respective supernatant was collected. Then, the absorbance of the supernatant was taken and quantified to determine the unentrapped drug by UV/Vis spectrometer at 341 nm. Finally, we calculated per cent drug loading and EE by following equations, and all the readings were taken in triplicate to minimize the error.

(1) $$\%\;\mathrm{Drug}\;\mathrm{loading}=\frac{\mathrm{Weight}\;\mathrm{of}\;\mathrm{drug}\;\mathrm{in}\;\mathrm{nanoparticles}}{\mathrm{Weight}\;\mathrm{of}\;\mathrm{nanoparticles}}\times 100$$

(2) $$\%\;\mathrm{EE}=\frac{\mathrm{Total}\;\mathrm{drug}-\mathrm{Unentrapped}\;\mathrm{drug}}{\mathrm{Total}\;\mathrm{drug}}\times 100$$

#### 2.4.3. In Vitro Drug Release with Dissolution Kinetics

We applied the dialysis tube method to estimate the in vitro release of drugs from AMX@Ctr–mpHANCs at 50 rpm with a temperature of 37 ± 0.5 °C. To determine the pH-responsive release of the drug from nanocarriers, the dissolution medium of different pH values (2.0, 4.5, and 7.4) was used. After a pre-calculated time, we took the samples with maintaining the sink condition. These samples were quantified by taking the absorbance at 341 nm. The amount of drug released from AMX@Ctr–mpHANCs is determined by putting each sample’s absorbance value in the standard curve equation. In this way, we calculated the total amount of drug that was released from drug-loaded NCs. Different models like first and zero-order, Korsmeyer-Peppas, and Higuchi models were applied in determining the dissolution pattern of AMX@Ctr–mpHANCs [22,23]. The software used for the analysis of these models was DDSolver, which is an MS Excel extension.

### 2.5. In Vitro Hemolytic Study

The blood samples were obtained from healthy persons with their consent. These samples were diluted with AMX@Ctr–mpHANCs (1%) and, subsequently, incubated for a specified time at 37 °C. We used unexposed samples (negative control), SDS 1% (positive control), and distilled water (vehicle control) as controls to compare the results. Afterwards, the incubated samples’ centrifugation was done at 6000 rpm for 6 min, and the supernatant was quantified through Nano-Drop (Thermo Scientific, Waltham, MA, USA) at 540 nm. Finally, a comparison of treated and untreated cells was made to estimate hemolysis (%).

### 2.6. Antibacterial Activity

Antibacterial potential of sole and AMX@Ctr–mpHANCs were estimated by applying broth dilution method, and colony-forming unit (CFU) assay. The bacteria were grown-up in a broth till the exponential growth stage had been attained. The turbidity of the bacteria was calculated by determining the bacterial suspension optical density. McFarland solution was used to compare the recorded bacterial turbidity [24]. The broth and bacterial culture were mixed, and AMX@Ctr–mpHANCs concentrations were loaded along with the inoculated broth. These sample tubes were incubated for a whole night in an incubator with continuous shaking at 37 °C. After 24 h, the samples were taken and measured for optical density (OD) of bacterial density through Elisa multiplate reader at 595 nm [25,26]. The comparison study involved positive (broth with culture) and negative (broth alone) controls. We also performed the CFU test to support the consistency in the results. The prepared formulations of AMX@Ctr–mpHANCs were patterned on the agar plate incubated. The bacterial colonies were calculated after 48 h of incubation [27].

### 2.7. Statistical Analysis

We performed all the experiments in triplicates to reduce the error and reported the mean ± standard deviation (SD). The comparison was necessary for the results, *t*-test and ANOVA were used to determine the significance level with a confidence interval of 95%.

## 3. Results and Discussion

The citrate functionalized mesoporous hydroxyapatite nanocarriers (Ctr–mpHANCs) were synthesized by core-shell technique and characterized for structural morphology, zeta size with potential, phase purity, carrier-drug interaction, surface area with porosity, TGA, and DSC.

### 3.1. Encapsulation Efficiency of AMX Inside AMX@Ctr–mpHANCs

We observed that encapsulation efficiency of AMX inside the AMX1@Ctr–mpHANCs, AMX2@Ctr–mpHANCs and AMX3@Ctr–mpHANCs is 89.13%, 91.05%, and 93.88%, respectively, as shown in Table 1. It is proposed that high encapsulation efficiency values (%) are due to mesoporous structure of hydroxyapatite nanocarriers that enables to load more drug molecules. It is also noticed that encapsulation efficiency directly depends on the concentration of the drug, i.e., increase the drug amount leads to increased encapsulation efficiency. Our study values are comparable with the reports on calcium phosphate nanocarriers [28,29].

### 3.2. Morphology and DLS Study

**Figure 1:**
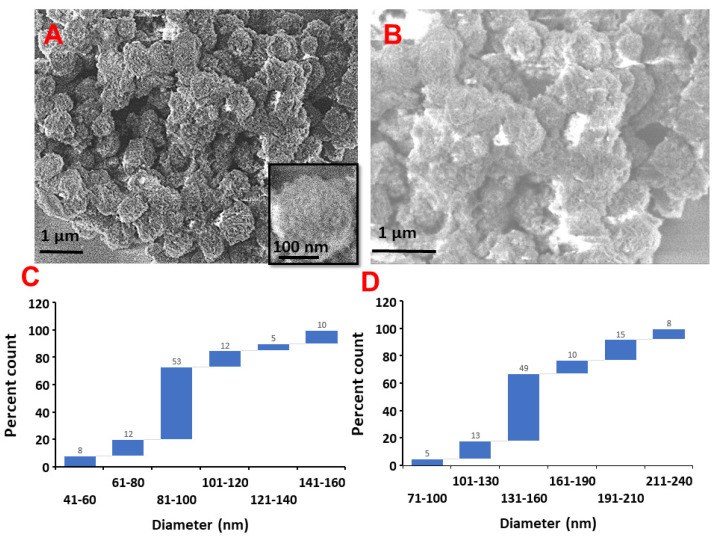
SEM images and size determination with per cent count (by using ImageJ software created by Wayne Rasband, retired from National institute of Health, with a permissive BSD-2 license, Madison, WI, USA) of (**A**,**C**) Ctr–mpHANCs (**B**,**D**) mpHANCs, respectively.

**Figure 2:**
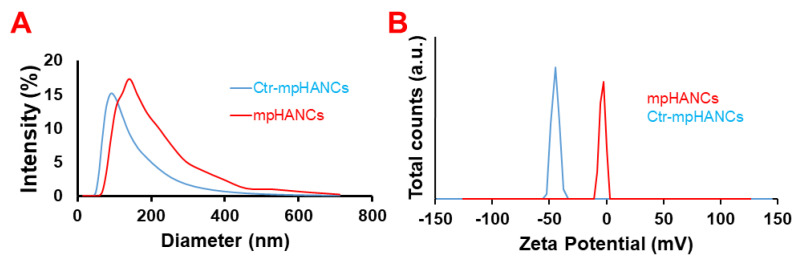
Zeta size (**A**) and zeta potential (**B**) of mpHANCs and Ctr–mpHANCs.

The morphology of Ctr–mpHANCs was examined with a scanning electron microscope (SEM). The SEM images in Figure 1A describe the roughly spherical and porous nature of Ctr–mpHANCs at low magnification and high magnification (Inset), while Figure 1B presents the SEM scan of non-functionalized mpHANCs. We assumed that Ctr–mpHANCs pores are responsible for high drug loading and sustained behaviour as shown in the BET study. Figure 1C,D presents the particle size (nm) of Ctr–mpHANCs and mpHNACs determined from SEM images by ImageJ software, respectively. The nanocarriers size correlates with the determined by zeta studies as shown in Figure 2A that shows the hydrodynamic zeta size of Ctr–mpHANCs and mpHANCs of about 91 nm and 142 nm, respectively. The zeta potentials of non-funtionalized mpHANCs and functionalized Ctr–mpHANCs are detailed in Figure 2B. We noticed that Ctr–mpHANCs have more zeta potential (−45 mV) as compared to mpHANCs (−3 mV), confirming that Ctr–mpHANCs is more stable formulation. We suggest that an increased zeta potential of Ctr–mpHANCs is due to the functionalization of citrate ions.

### 3.3. Phase Determination and Drug-Nanocarriers Interaction

**Figure 3:**
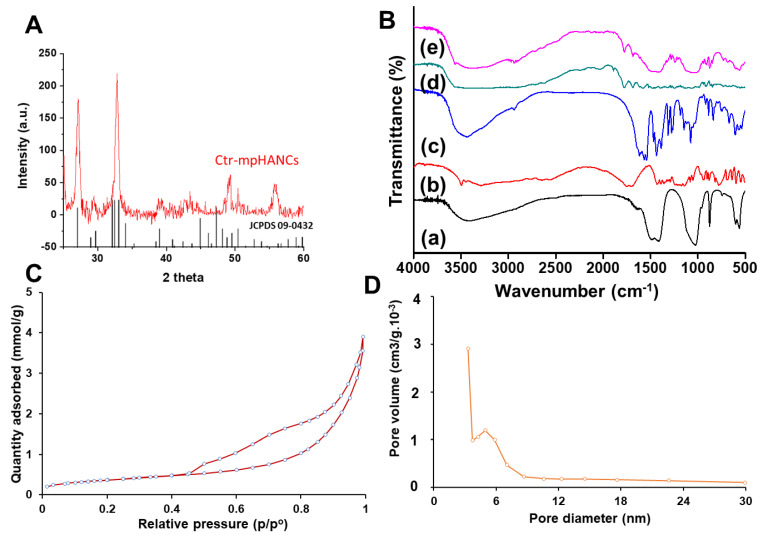
(**A**) XRD patterns of hydroxyapatite (JCDPS file no. 09-0432) and Ctr–mpHANCs; (**B**) FTIR pattern of (a) mpHANCs, (b) citric acid, (c) Ctr–mpHANCs, (d) Amoxicillin, and (e) AMX@Ctr–mpHANCs; (**C**) N_2_-adsorption/desorption isotherm of Ctr–mpHANCs; and (**D**) BJH distribution of pore size of Ctr–mpHANCs.

XRD peaks of Ctr–mpHANCs are presented in Figure 3A and confirmed the hydroxyapatite phase of the prepared sample as the peaks data matches with the JCPDS standard data of hydroxyapatite (File number: 09-0432). It indicates the crystalline phase of hydroxyapatite material with wide-ranging peaks at 30–35° [29,30]. The major XRD peaks at 25.29°, 29.38°, 31.97°, 32.92°, 33.32°, and 33.8° attributed to (002), (102), (210), (211), (112), and (300) planes of HA, correspondingly [31]. Scherrer equation was used to calculate the Ctr–mpHANCs crystallite size and was found to be approximately 82 nm [32].

FTIR study confirmed the stabilization of hydroxyapatite with citric acid, drug integrity inside nanocarriers, and possible interaction of the drug with mpHANCs. FTIR spectrum of mpHANCs, citric acid, and amoxicillin can be seen in Figure 3B. The peaks of the –OH group and PO_4_^−3^ bands recognized the HA specificity. At the same time, citric acid spectra reveal the distinct bands at 3444.29 cm^−1^, 1729.60 cm^−1^, and 1000–1500 cm^−1^, describing hydroxyl bending, stretching of C=O, and vibrations of C–C, C–O, and C–OH, respectively [33]. Figure 3B(c) confirms the citrate stabilization of hydroxyapatite by indicating the main peaks of hydroxyapatite and citric acid. The prominent peaks of amoxicillin at 1772.54 cm^−1^, 1680.21 cm^−1^, and 1587.45 cm^−1^ can be seen in sole spectra of AMX and AMX loaded Ctr–mpHANCs [20]. This also endorses the loading of the drug in prepared formulations of AMX@Ctr–mpHANCs [34].

### 3.4. Surface Area and Porosity

We determined the specific surface area (SSA) of Ctr–mpHANCs with porous stuff like pore cumulative volume and pore width through N_2_-physisorption isotherm, as shown in Figure 3C. According to IUPAC guidelines, the prepared NSs have shown isotherm (type-IV) and hysteresis loop (H3) [35]. We found the BET specific surface area and pore diameter of about 182.35 m^2^/g and 4.2 nm, respectively, signifying a narrow distribution pattern of pore width. It was noticed that Ctr–mpHANCs had a more significant surface area compared to the previous reports of hydroxyapatite [36,37]. We also observed the phenomenon of pore condensation in the obtained isotherms, which indicate that at p (pressure) lower than p_0_ (saturation pressure) of liquid, gas may be condensed from a liquid-like phase to a pore [38]. It was assumed that Ctr–mpHANCs have a higher surface area due to the lesser size of NCs crystallite as diffraction peaks have proposed this hypothesis and these results are comparable to the previous reports [39,40]. IUPAC states that a wide-ranging hysteresis loop denotes the shape of the pores like the bottle’s neck, and this point was sustained with the study of Barrett–Joyner–Halenda (BJH) adsorption and desorption dv/dw pore volume (Figure 3D). This graph specifies delayed desorption due to increased and decreased pore width during adsorption and desorption, respectively [41]. This study may suggest that prolonged or sustained drug release from Ctr–mpHANCs is due to these connected pores.

### 3.5. Physical State and Thermal Behaviour of Amoxicillin inside Ctr–mpHANCs

**Figure 4:**
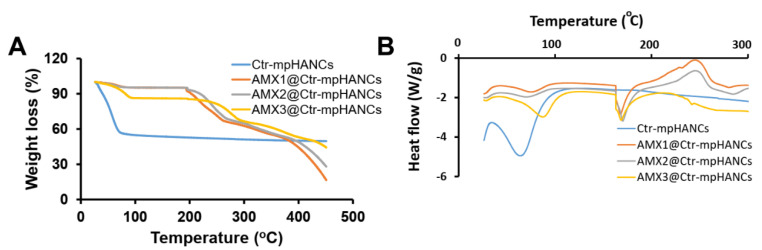
(**A**) TGA and (**B**) DSC of sole and AMX-loaded Ctr–mpHANCs.

The thermal stability of sole and drug-loaded Ctr–mpHANCs was evaluated by TGA, as shown in Figure 4A. We found a significant weight loss in sole Ctr–mpHANCs below 100 °C that may be due to the removal of adsorbed water. After this, there is no significant weight loss till 450 °C. In AMX@Ctr–mpHANCs, significant weight loss was seen from 100 °C to 450 °C and was supposed to be due to amoxicillin denaturation. We observed that weight loss is decreased as the drug concentration increases, and it may be due to the strong binding of the drug. DSC confirm the physical state of AMX inside all the formulations, as shown in Figure 4B. DSC graphs showed the endothermic peak of amoxicillin at 184 °C corresponding to its melting point, which can be seen in amoxicillin loaded Ctr–mpHANCs. Amoxicillin peak is not suppressed after loading in Ctr–mpHANCs, describing the crystalline nature of the drug [34].

### 3.6. In Vitro Drug Release

**Figure 5:**
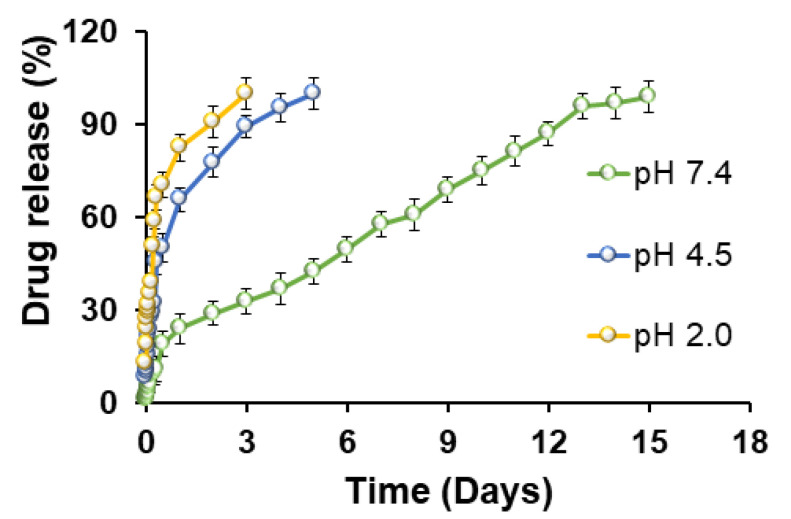
Drug release profile of AMX@Ctr–mpHANCs at pH 2.0, 4.5, and 7.4.

The per cent AMX release from AMX@Ctr–mpHANCs is studied at different pH as shown in Figure 5. In microbial infections, the bone tissues pH drops down, and it is necessary to evaluate the drug release at different pH levels. The dialysis tube method was used to calculate drug release, and AMX@Ctr–mpHANCs were placed in a dissolution medium of pH 2.0, 4.5 and 7.4 to see the drug release pattern. We noticed that almost 100% of the drug was released from the AMX@Ctr–mpHANCs within 3 d at pH 2.0, while at pH 4.5, the whole drug was released in 5 d. We found the sustained drug release behaviour for 15 d at pH 7.4. It is proposed that higher drug release at low pH is due to the delicate structure of mpHANCs and will benefit from treating the bacterial infection at low pH. The drug release kinetic models presented the following of Higuchi model and zero-order kinetic by AMX@Ctr–mpHANCs at pH 7.4. Likewise, the release exponent (n) value expresses the anomalous diffusion as per the Korsmeyer–Pappas model. On the other hand, at lower pH, the AMX release from AMX@Ctr–mpHANCs has followed fickian diffusion as described by Korsmeyer–Pappas. This study reveals better results in comparison to previous studies [42,43,44].

### 3.7. In Vitro Hemolysis

**Figure 6:**
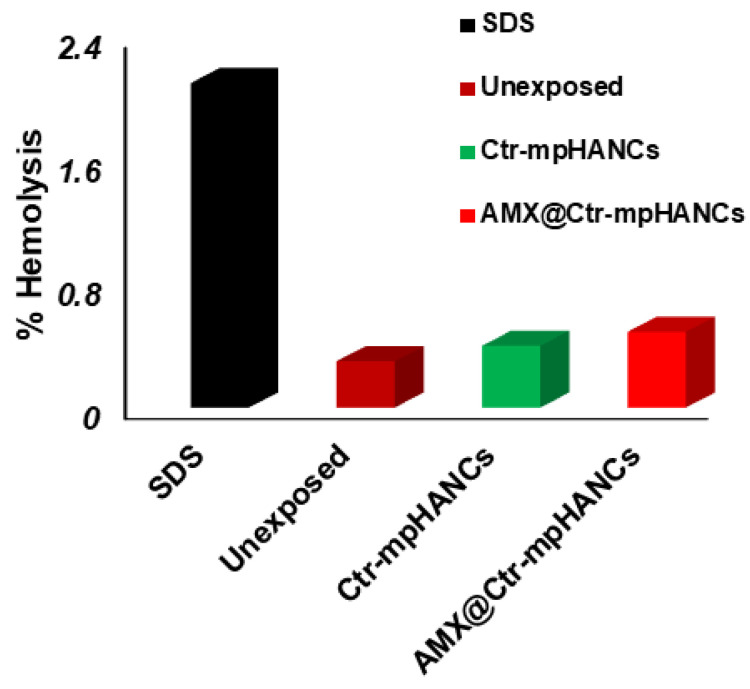
Per cent hemolysis study to confirm no harmful effect of our formulation on RBCs.

After in vivo administration, plasma proteins and RBCs are the physiological constituents with which drug/NCs interact. Because of this, it is critical to check the interaction of the blood with Ctr–mpHANCs in a specific environment. Figure 6 showed that there is no human RBCs hemolysis byAMX@Ctr–mpHANCs. As per standards of ASTM 756-00, the ratio of hemolysis lower than 2 is termed as a non-hemolytic value. The hemolysis-free nature of Ctr–mpHANCs is due to its similarity with natural bone structure [45]. Moreover, Ctr–mpHANCs does not cause hemolysis because its charged surface sites avoid interaction with RBC [46].

### 3.8. Resistance of Microbial Strains

It was necessary to determine the resistance pattern of microbial cultures before using for in vitro evaluation of sole Ctr–mpHANCs and AMX@Ctr–mpHANCs. The disk diffusion method was performed to assess the antibacterial activities of standard disks of different antibiotics as shown in Table 2. It was noticed that ceotaxime, ceftriaxone, and amoxicllin have showed resistance against *MRSA*, *P. aeruginosa* and *E. coli*. We used amoxicllin as a model drug due to its wide range resistance against bacteria, narrow spectrum, unable to resist beta-lactamases and easy availability to us.

### 3.9. Antibacterial Activities

**Figure 7:**
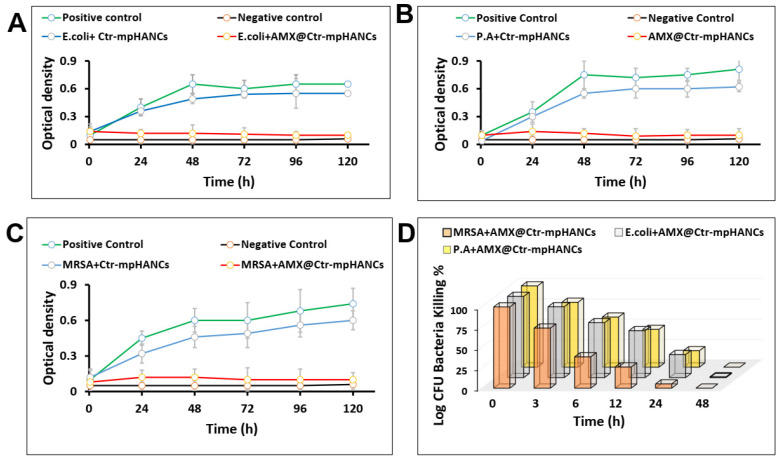
Bacterial kinetics growth of (**A**) *E. coli*; (**B**) *P. aeruginosa* (P.A); (**C**) *MRSA* in presence of sole Ctr–mpHANCs and AMX@Ctr–mpHANCs; and (**D**) CFU assay showing the antibacterial potential of AMX@Ctr–mpHANCs against *MRSA*, *E. coli*, and *P. aeruginosa*.

Finally, we selected the formulation with higher % encapsulation efficiency, AMX3@Ctr–mpHANCs, to evaluate the antibacterial potential against *P. aeruginosa*, *E. coli*, and *MRSA*, as shown in Figure 7. AMX@Ctr–mpHANCs had a significant contribution towards antibacterial activity due to strong adhesion and adsorption ability on the bacterial cell wall [47]. Therefore, it is proposed that AMX@Ctr–mpHANCs kill the bacteria by interacting with the bacterial surface [48]. In addition, scientists report that strong adhesion/adsorption of material with bacteria results in decreased bacterial growth by rupturing the cell wall [49,50].

We observed that sole NCs were unable to kill the bacteria. We performed two tests to know the antimicrobial potential of AMX@Ctr–mpHANCs, i.e., broth dilution and CFU. Figure 7A–C shows the growth pattern of *P. aeruginosa*, *E. coli*, and *MRSA* after treatment with sole Ctr–mpHANCs and AMX@Ctr–mpHANCs. We observed a significant reduction in the bacterial growth with AMX@Ctr–mpHANCs and no bacteria-killing with sole Ctr–mpHANCs. The positive and negative controls are inoculated culture broth without NC/drug and nutrient broth alone, respectively. Figure 7D denotes the CFU assay by determining the per cent bacteria-killing over time [51]. The sole NCs and positive controls showed bacterial growth and confirmed their role as a carrier only. In vitro release profile depicts an increased AMX release with passage of time. Similary, the CFU assay results show that as time passes, the bacterial growth decreases. So, we suggest an inverse relation between AMX release from AMX@Ctr–mpHANCs and its antibacterial action. Our study proposed the role of Ctr–mpHANCs to deliver the drug to the target site infected by the microbes.

## 4. Conclusions

Our study proposed the synthesis of citrate functionalized mesoporous HANCs with the core-shell technology. We observed substantial loading of the drug inside the Ctr–mpHANCs, and it may be due to the porous structure of NSs. We report the pH-triggered drug release from Ctr–mpHANCs and suggest the drug’s immediate release and sustained release at acidic and basic pH, respectively. We know that in the bone associated infections caused by bacteria, the pH drops to an acidic level. So, the acid-labile nature of HANCs causes their degradation, and the loaded drug is released to kill bacteria. In conclusion, we suggest Ctr–mpHANCs as a therapeutic drug model carrier to deliver to the target site.

## Figures and Tables

**Table 1:** Encapsulation efficiency (%) of the formulations (n = 3, mean ± SD).

Formulation Code	Drug Loading Concentration	Encapsulation Efficiency (%)
AMX1@Ctr–mpHANCs	20 mM	89.13 ± 1.05
AMX2@Ctr–mpHANCs	30 mM	91.05 ± 1.51
AMX3@Ctr–mpHANCs	50 mM	93.88 ± 1.42

**Table 2:** Determining the resistance pattern of different antibiotic discs.

Antibiotic Disc (Unit µg)	Inhibition Zone (mm)		
	*MRSA*	*P. aeruginosa*	*E. coli*
CTX (30)	No	No	No
CRO (30)	No	No	No
ATM (30)	No	No	16
AMX (25)	No	No	No
AMC (10)	17	No	No
IPM (10)	17	13	27
CAZ (30)	No	No	18
VA (30)	19	No	No

## Data Availability

Data sharing is not applicable.
